# The Protective Effect of 11-Keto-β-Boswellic Acid against Diabetic Cardiomyopathy in Rats Entails Activation of AMPK

**DOI:** 10.3390/nu15071660

**Published:** 2023-03-29

**Authors:** Jozaa Z. AlTamimi, Nora A. AlFaris, Ghedeir M. Alshammari, Reham I. Alagal, Dalal H. Aljabryn, Mohammed Abdo Yahya

**Affiliations:** 1 Department of Physical Sports Sciences, College of Education, Princess Nourah Bint Abdulrahman University, P.O. Box 84428, Riyadh 11671, Saudi Arabia; 2 Department of Food Science and Nutrition, College of Food and Agricultural Sciences, King Saud University, P.O. Box 84428, Riyadh 11451, Saudi Arabia; 3 Department of Health Sciences, College of Health and Rehabilitation Sciences, Princess Nourah Bint Abdulrahman University, P.O. Box 84428, Riyadh 11671, Saudi Arabia

**Keywords:** 11-keto-β-boswellic acid, diabetic cardiomyopathy, streptozotocin, oxidative stress, AMPK, Nrf2

## Abstract

This study examined the protective effect of 11-keto-β-boswellic acid (AKBA) against streptozotocin (STZ)-induced diabetic cardiomyopathy (DC) in rats and examined the possible mechanisms of action. Male rats were divided into 5 groups (n = 8/each): (1) control, AKBA (10 mg/kg, orally), STZ (65 mg/kg, i.p.), STZ + AKBA (10 mg/kg, orally), and STZ + AKBA + compound C (CC/an AMPK inhibitor, 0.2 mg/kg, i.p.). AKBA improved the structure and the systolic and diastolic functions of the left ventricles (LVs) of STZ rats. It also attenuated the increase in plasma glucose, plasma insulin, and serum and hepatic levels of triglycerides (TGs), cholesterol (CHOL), and free fatty acids (FFAs) in these diabetic rats. AKBA stimulated the ventricular activities of phosphofructokinase (PFK), pyruvate dehydrogenase (PDH), and acetyl CoA carboxylase (ACC); increased levels of malonyl CoA; and reduced levels of carnitine palmitoyltransferase I (CPT1), indicating improvement in glucose and FA oxidation. It also reduced levels of malondialdehyde (MDA); increased mitochondria efficiency and ATP production; stimulated mRNA, total, and nuclear levels of Nrf2; increased levels of glutathione (GSH), heme oxygenase (HO-1), superoxide dismutase (SOD), and catalase (CAT); but reduced the expression and nuclear translocation of NF-κB and levels of tumor-necrosis factor-α (TNF-α) and interleukin-6 (IL-6). These effects were concomitant with increased activities of AMPK in the LVs of the control and STZ-diabetic rats. Treatment with CC abolished all these protective effects of AKBA. In conclusion, AKBA protects against DC in rats, mainly by activating the AMPK-dependent control of insulin release, cardiac metabolism, and antioxidant and anti-inflammatory effects.

## 1. Introduction

The heart is the most known metabolically flexible (omnivore) organ that can switch between several oxidative substrates to meet its high requirement of ATP [1]. However, this depends on the cardiac activity (workload), as well as substrate and insulin availability [1]. In the normal heart, fatty acids (FAs), glucose, and ketone bodies are the major energy substrates and provide 40–60%, 20–40%, and 10–20% of the overall ATP, respectively [2]. This balance is usually met by the action of insulin, which can stimulate glycolysis and inhibit FAs oxidation, despite increasing FFA uptake [3]. In the absence of insulin, substrate flexibility is impaired, and the heart modifies its energy metabolism by augmenting free fatty acid (FFA) oxidation and suppressing glucose utilization, glycolysis, and oxidation, allowing glucose to enter non-ATP-producing reactions [4]. These factors can lead to cardiomyocyte injury by promoting the production of ROS and inflammatory cytokines, which further triggers cardiac oxidation, inflammation, fibrosis, and apoptosis [5,6,7,8,9,10]. As a result, cardiac contractility, as well as the systolic and diastolic functions are compromised, leading to a clinical condition known as diabetic cardiomyopathy (DCM) [5,6]. Indeed, diabetic cardiomyopathy is the most commonly known complication in patients with type 1 and type 2 diabetes mellitus (DM) and is the most significant contributing factor to the increased mortality among them [5].

DCM also involves alternating energy and metabolic-specific signaling pathways [11]. The adenosine monophosphate-activated protein kinase (AMPK) is the major energy sensor molecule in the majority of cells that can regulate substrate metabolism and stimulate ATP production and cell survival in response to high AMP or ADP/ATP ratio [12]. Even though AMPK is a novel regulator of cellular energy sensors, it functions as an important therapeutic target for obesity, metabolic disease, and DM by acting through defined metabolic pathways in the liver, adipose tissue, and muscles [13]. These mechanisms include inhibiting gluconeogenesis, stimulating glucose intake and FA oxidation, and suppressing lipogenesis [14,15]. Studies have also shown a potent protective effect of AMPK against diabetes and cardiovascular health via the regulation of glucose uptake and utilization, lipid synthesis, fatty acid oxidation, autophagy, oxidative stress, inflammation, and apoptosis [16]. Indeed, the activities of AMPK are significantly depleted in the heart of diabetic animals (i.e., T1/T2DM) and are associated with cardiomyocyte lipotoxicity, oxidative stress, inflammation, reduced contractility, apoptosis, and remodeling [17,18,19]. In addition, genetic depletion or pharmacological inhibition of AMPK-induced structural and functional disturbances, remodeling, and dilated cardiomyopathy have been observed in experimental healthy mice [20,21]. On the contrary, stimulating cardiac AMPK activities in diabetic or failing hearts has been reported to preserve LV structure and mitochondrial function and augmented contractility by decreasing ROS generation, suppressing inflammatory cytokine production, promoting autophagy, enhancing antioxidant capacity, and improving glucose utilization [16,17,18,22,23,24,25]. Therefore, much attention is being currently given to searching for natural safe compounds that can attenuate DM and its complications by activating AMPK [16].

Plant flavonoids are widely used to treat DM and its complications, as they are devoid of major toxicities and have excellent antioxidant and anti-inflammatory potentials [26]. *Boswellia serrata* (*B. serrata*) is a natural gum resin, largely grown in the Middle East and Africa, and has been traditionally used to treat several chronic disorders [27,28]. 3-O-acetyl-11-keto-β-boswellic acid (AKBA) is the major boswellic acid isolated from *B. serrata* [28]. Accumulating experimental and clinical data attest to the exceptional ability of AKBA to treat several disorders, including infection, cancer, sepsis, hypertension, arthritis, liver and brain damage, memory deficits, and colitis due to its potent antioxidant and anti-inflammatory effects [28,29,30,31,32,33]. In addition, studies have shown that AKBA can act on several cellular targets, including transcription and growth factors, as well as signaling mediators and pathways [28]. Moreover, treatment with *B. serrata* extracts of AKBA exhibits antidiabetic and wound-healing properties. Both can attenuate hepatic and pancreatic damage through hypoglycemic, antihyperlipidemia, antioxidant, and anti-inflammatory properties effects [34,35,36,37]. Interestingly, some previous studies have also shown the exceptional ability of AKBA to modulate AMPK activity in various tissue. Within this context, it has been documented that AKBA protects against rotenone-induced neurotoxicity and breast carcinoma by activating AMPK [38,39].

Streptozotocin (STZ) is the most commonly known chemical that can induce insulin deficiency in animals by oxidative damage of the pancreatic β-cells to produce a phenotype similar to T1DM. STZ-diabetic rats and mice have been widely used to study the pathogenesis of DCM associated with T1DM, as well as the effectiveness of therapeutic drugs and plant-derived compounds [40,41,42]. To date, no single study has yet examined the cardioprotective effect of AKBA in diabetic animal models, making area of research interest. Therefore, in this study, we first aimed to examine if chronic treatment with AKBA can attenuate cardiac damage and injury in STZ-diabetic rats (STZ). In addition, and based on the above-mentioned studies demonstrating the antidiabetic, antioxidant, and anti-inflammatory effects of AMPK as well as the ability of AKBA to regulate this molecule, we tested the hypothesis that the cardioprotective effect of AKBA in this animal model is mediated by the activation of AMPK.

## 2. Materials and Methods

### 2.1. Animals

Wistar rats were used in this study. All rats were provided and maintained with care by the Experimental Animal Care Center at King Saud University, Saudi Arabia. All selected rats were initially males and weighed 160 g ± 20 g. Consistently, all rats were housed under a 12 h dark–light cycle at room temperature. All experimental procedures performed in this study were approved by the official review board at Princess Nourah University, Riyadh, KSA (IRB Number 20-0096), which follows the guidelines established by the US National Institutes of Health [43].

### 2.2. Chemicals and Drugs

STZ powder (Cat. # S0130), AKBA, compound C (CC) (an AMPK inhibitor) (Cat # 171260), and dimethyl sulfoxide (DMSO) (Cat # A9855, Cat #, respectively) were purchased from Sigma Aldrich (St. Louis, MO, USA). An assay kit to measure levels of malondialdehyde (MDA) (Cat. # MBS268427) and activities of acetyl CoA carboxylase (ACC) (Cat. # MBS8303295), as well as an ELISA kit to measure the levels of carnitine palmitoyltransferase I (CPT1) (Cat. # MBS2602676), troponin-1 (Tpn1) (Cat. # MBS269777), creatinine kinase-MB (CK-MB) (Cat # MBS2019791), free fatty acids (FFAs) (Cat. No. # MBS014345), Bcl2 (Cat. # MBS2881713), Bax (Cat. # MBS935667), caspase-3 (Cat. # MBS018987), and cytochrome-c (Cat. # MBS9304546) were provided by MyBioSource (San Diego, CA, USA). An assay kit to measure plasma glucose concentration (Cat. # 81695) was purchased from Chrystal Chem (Houston, TX, USA). An ELISA kit to measure plasma insulin levels (Cat. # ERINS) was provided by Thermo Fisher (Waltham, MA, USA). An assay kit to quantify the concentrations of triglycerides (TGs) (Cat. # ECCH-100) was provided by BioAssay Systems (Hayward, CA, USA). Assay kits to measure levels of total cholesterol (Cat. # 10009582) and low-density lipoproteins (LDL-c) (Cat. # 79960) were purchased from Crystal Chemicals, TX, USA, respectively. ELISA kits to measure levels of MyBioSource, CA, USA). ELISA kits to measure the total levels of total superoxide dismutase (SOD) (Cat. # RTFI00215), tumor necrosis factor-alpha (TNF-α) (Cat # RTFI01177) glutathione peroxidase (GPX) (Cat. No, RTEB0206), interleukine-6 (IL-6) (Cat# RTEB0061), and glutathione (GSH) (Cat # RTEB1811) were supplied from Assay Genie (London, UK). An ELISA kit for the activity/phosphorylation of AMPK (Thr 172) (Cat. # KHO0651) was purchased from Thermo Fisher (Warrington, UK). A colorimetric kit to quantify the activity/phosphorylation of phosphofructokinase (PFK) (Cat. No. K776) was purchased from BioVision (Milpitas, CA, USA). An ELISA kit to measure the levels of pyruvate dehydrogenase (PDH) (Cat. #ab287837) was purchased from Abcam (Cambridge, UK). An assay kit to measure levels of ATP (Cat. # A22066) was supplied by ThromoFisher (Waltham, MA, USA). A mitochondria isolation kit (Cat. # ab110168) and a cytoplasm/nuclear fraction isolation kit (Cat # Ab113474) were supplied by Abcam (London, UK). ELISA kits for the assessment of levels of erythroid 2-related factor 2 (Nrf2) (Cat. # 50296) and nuclear factor kappa-beta (NF-κB) (Cat. # 31102) were supplied by Active Motif (Tokyo, Japan). An RNA isolation kit (Cat. # 74004) was provided by Qiagen (Hilden, Germany). A cDNA synthesis kit (Cat. # K1621) was provided by Thermo Fisher. A Ssofast Evergreen Supermix kit (Cat. No. 172-5200) was purchased from (BioRad, Hercules, CA, USA).

### 2.3. STZ-Induced T1DM

Induction of T1DM followed the procedure previously reported in our laboratory, with a single dose of STZ being used [44]. In brief, STZ was freshly prepared in sodium citrate buffer (pH = 5.5) and administered intraperitoneally (i.p.) to all selected rats at 7:00 a.m. at a final concentration of 65 mg/kg. Fasting blood glucose levels in all STZ-treated were measured using a commercial glucometer 1-week post-STZ injection, and those with values higher than 320 mg/dl were selected as T1DM and were included directly in the further experiments.

### 2.4. Experimental Design

**Figure 1:**
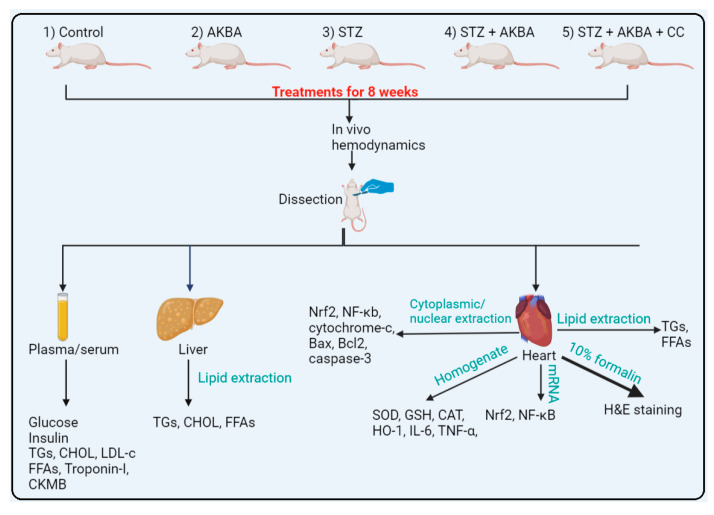
The experimental design used in the study.

AKBA and CC were dissolved in DMSO and diluted to the desired concentration, with the DMSO percentage being 0.1%. Age-matched rats with preestablished diabetes and nondiabetic rats were randomly selected and distributed to 8 rats per groups. STZ-diabetic rats were selected randomly and segregated into the following groups (n = 8 rats/group): (1) control group, only and orally treated with the vehicle (0.5 mL 0.1% DMSO, orally); (2) AKBA-treated group, nondiabetic rats orally administered 0.5 mL AKBA solution (10 mg/kg, orally); (3) STZ-treated group, rats already diagnosed with T1DM and orally administered 0.5 mL of 0.1% DMSO; (4) STZ + AKBA-treated group, rats already diagnosed with T1DM and orally administered 0.5 mL of AKBA solution (10 mg/kg, orally); and (5) STZ + AKBA + compound C (CC)-treated group, rats with T1DM e treated with 0.5 mL of dissolved CC solution (0.2 mg/kg, i.p.) and then 0.5 mL of AKBA solution (10 mg/kg). All treatment was given daily for 8 weeks. Treatment with CC was given 6 h before treatment with AKBA (Figure 1).

### 2.5. Regimen and Dose Selections

All experiments were conducted for 8 weeks, as described elsewhere, as a minimum period to establish DCM in rats associated with oxidative stress, inflammation, fibrosis, and apoptosis [45]. The selected dose of AKBA was based on other in vivo studies that demonstrated hypoglycemic and pancreatic protective effects in STZ-treated mice. The same dose demonstrated hypoglycemic, hypolipidemic, hepatic, pancreatic, and renal antioxidant effects in high-fat-diet (HFD)-fed rats [37]. The dose and in vivo administration of CC were based on the studies of others [46,47].

### 2.6. Collection of Plasma and Biochemical Measurement

On the last day of the experiment, all rats fasted overnight. The next day, all rats were anesthetized using a single i.p. injection with ketamine/xylazine mixture (80/10 mg/mg), and blood from their sample was collected in EDTA or gel-containing plain tubes. These tubes were then centrifuged at 500× *g* for 20 min at room temperature to collect both plasma and serum, which were all stored at −20 °C. Plasma levels of fasting glucose and insulin levels, as well as serum levels of troponin-1 (Tpn1), creatinine kinase-MB (CK-MB), triglycerides (TGs), cholesterol, FFAs, and low-density lipoproteins (LDL-cs), were measured using the provided assay kits. All measurements were performed for n = 8 samples/groups as instructed by each kit provided by the manufacturers.

### 2.7. Measurement of Cardiac Function

After blood collection, all rats were returned to their cages. Three days later and over 10 days, cardiac function in all groups of rats was assessed as previously described in other laboratories and by others using a precalibrated Millar pressure catheter (model # SPR-320) [47,48,49]. In brief, rats were anesthetized using a mixture of ketamine/xylazine (80/10 mg/mg) and were in a supine position on a heating table. A thermal probe was anally inserted, and the temperature was maintained at 37 °C. In addition, eye ointment was applied to both ears during the protocol to reduce dehydration. After anesthesia, it was confirmed that the carotid artery was located, ligated, and opened, and the pressure catheter was forwarded to the left ventricle (LV). The catheter was connected to an amplifier that was itself connected to a PowerLab data acquisition system (model 8/35, AD Instrument, Sydney, Australia). After stabilization, the signal was recorded for 10 min and then analyzed using LabChart software (version 8 AD Instrument, Sydney, Australia). The signal was analyzed, and the following parameters of the LVs were used: (1) dP/dt_max_ (maximum increase in pressure overtime during isovolumetric contraction) and (2) LVSP (left ventricular systolic pressure)—as markers of cardiac contractility and systolic function—as well as (3) dP/dt_min_ (the minimum pressure in the LV over time during isovolumetric relaxation) and (4) LVEDP (left ventricular end-diastolic pressure)—as markers of LV relaxation and diastolic function.

### 2.8. LV Collection and Processing

After hemodynamic measurements, animals of all groups were killed via cervical dislocation. The heart and liver of each rat were collected and placed on ice, and the LVs were separated. Livers and LVs were cut into smaller sections, some of which were preserved in 10% formalin for histological evaluation, whereas the remaining were frozen at −80 °C for further processing.

### 2.9. Serum and LV Lipid Measurement

The cardiac lipid fraction was isolated from the frozen heart tissue levels as initially reported by Folch et al. [50] and as described in more detail in our previous reports [47]. The LV levels of TGs and FFAs were determined with the same kits used to measure their levels in the serum.

### 2.10. Analyses in LV Tissue

Parts of the frozen LV tissues were homogenized in ice-cold neutral phosphate-buffered saline (PBS) to prepare tissue homogenates. The homogenates were centrifuged at 12,000× *g* for 20 min at 4 °C to collect the supernatants containing all proteins. Homogenates were preserved at −80 °C and used later to evaluate the activity of ACC, AMPK, and PFK, PDH, as well as the levels of malondialdehyde (MDA), SOD, TNF-α, GPX, IL-6, GSH, ACC, CPT1, and ATP using the available kits mentioned. ATP levels in all homogenates were measured using a colorimetric kit (Cat. No A22066, Invitrogen/Molecular Probes). Moreover, cytoplasmic and nuclear levels of the erythroid 2-related factor 2 (Nrf2) and nuclear factor kappa-beta (NF-κB) in the frozen cytoplasmic and nuclear fractions, as well as levels of Bcl2, Bax, caspase-3, and cytochrome-c in cytoplasmic fractions, were measured using the provided kits. All measurements were conducted as instructed by each respective kit and for n = 8 samples/group.

### 2.11. Measurement of the Mitochondria Permeability Transition Pore Potential (mtPTP) Opening

Measurement of mtPTP in the isolated mitochondria was performed as described by others [51]. The principle of the method relies on the fact that the opening of mitochondria is induced by high calcium (Ca^2+^) levels and is accelerated by the presence of oxidative stress. Briefly, freshly isolated mitochondria were first suspended in a respiratory media containing succinate (5 mM), sucrose (71 mM), mannitol (215 mM), and HEPES (3 mM) and then stimulated with CaCl_2_ (400 µM) and tert-butyl hydroperoxide (75 µM). The reduction in absorbance over 15 min, as a marker of mtPTP opening, was monitored using a spectrophotometer. Analysis was performed for 8 samples/group as per the kits’ instructions.

### 2.12. Real-Time PCR

Total RNA was isolated, and the first strand of cDNA was prepared using the provided kits. Primer pairs for the amplification of AMPKα, Nrf2, NF-κB, and the reference gene (β-actin) were synthetized and supplied by Thermo Fisher, USA, based on studies of others in rats [47,52]. Amplification reactions were performed using a CFX96 PCR machine with the Ssofast Evergreen Supermix kit. Amplification followed the kit instruction’s with the following program: 1 heating cycle for 30 s at 98 °C; 40 cycles of denaturation (98 °C) and annealing/extension (60 °C), each of 5 s; and a final melting step for 5 s at 95 °C. The relative mRNA level of each of these targets was normalized to its level of β-actin using the 2ΔΔCT method, and the data are presented as relative to the control group.

### 2.13. Histopathological Evaluation

LVs were collected into 10% buffered formalin for 24 h and then rehydrated in increasing concentrations of ethanol. All tissues were next cleared with xylene, embedded in paraffin, sectioned at 3–5 µm, and routinely stained with hematoxylin and eosin (HE). All slides were examined under a light microscope and photographed at 200×.

### 2.14. Statistical Analysis

All data were analyzed using one-way ANOVA with GraphPad Prism software (version 9). Normality was tested using the Kolmogorov–Smirnov test. The comparison between various groups was performed using Tukey’s post hoc test. Data were considered significantly different at *p* < 0.05.

## 3. Results

### 3.1. AKBA Activated AMPK, Improved Mitochondrial Function, and Increased ATP in the LVs of STZ-Diabetic Hearts

**Figure 2:**
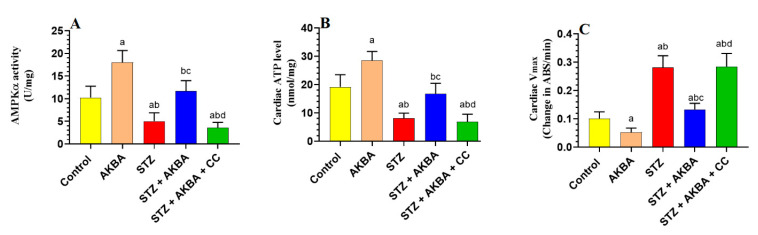
Activities of AMPKα (**A**), total levels of ATP (**B**), and the maximum reduction of Vmax as a marker of mitochondrial membrane potential (mtPTP) opening (**C**) in the left ventricles (LVs) of all rat groups. Data were analyzed with one-way ANOVA followed by Tukey’s test. Values are presented as mean ± SD (n = 8/group). Data were considered significantly different at *p* < 0.5. a: significantly different as compared to control rats; b: significantly different as compared to AKBA-treated rats; c: significantly different as compared to STZ-diabetic rats; d: significantly different as compared to STZ + AKBA-treated rats. CC—compound C (a selective AMPK inhibitor).

AMPK activities and total ATP levels were significantly decreased (*p* < 0.5), whereas reduction in absorbance (V_max_), as a marker of mtPTP opening, was significantly increased (*p* < 0.5) in the LVs of STZ-treated rats when compared to the control group rats (Figure 2A–C). The total activities of AMPK and total levels of ATP were significantly increased (*p* < 0.5), whereas values of V_max_ were significantly decreased in the LVs of the AKBA- and STZ + AKBA-treated rats as compared to the control and STZ-diabetic rats, respectively (Figure 2A–C). These effects were significantly reversed (*p* < 0.5) in the LVs of the STZ + AKBA + CC-treated rats as compared to the STZ + AKBA-treated rats (Figure 2A–C). No significant variations (*p* > 0.5) in the levels of any of these markers were seen in the STZ-diabetic rats compared to the STZ + AKBA + CC rats. These data suggest that AKBA enhances ATP levels and improves mitochondrial function via activating AMPK.

### 3.2. AKBA Stimulated Glycolysis and FA Oxidation in the LVs of STZ-Diabetic Rats by Stimulating AMPK

**Figure 3:**
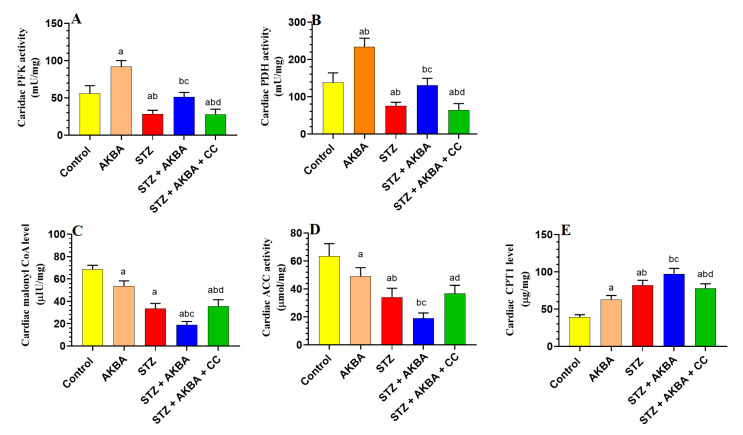
Activities of phosphofructokinase (**A**), pyruvate dehydrogenase (PDH) (**B**), levels of malonyl CoA (**C**), activities of acetyl CoA carboxylase (**D**), and levels of carnitine palmitoyltransferase I (CPT1) (**E**) in the left ventricles (LVs) of all groups of rats. Data were analyzed with one-way ANOVA followed by Tukey’s test. Values are presented as mean ± SD (n = 8/group). Data were considered significantly different at *p* < 0.5. a: significantly different as compared to control rats; b: significantly different as compared to AKBA-treated rats; c: significantly different as compared to STZ-diabetic rats; d: significantly different as compared to STZ + AKBA-treated rats. CC–compound C (a selective AMaPK inhibitor).

Activities of PFK, PDH, and ACC1, as well as the levels of malonyl CoA, were significantly reduced (*p* < 0.5), but levels of CPT1 were significantly increased *p* < 0.5) in the LVs of STZ-treated rats as compared to control rats, indicating increased FA oxidation and reduced glycolysis and glucose oxidation (Figure 3A–E). Activities of PFK and PDH, as well as levels of CPT1, were significantly increased (*p* < 0.5), whereas levels of malonyl CoA and activities of ACC1 were significantly decreased (*p* < 0.5) in the LVs of AKBA- and STZ + AKBA-treated rats as compared to control and STZ-diabetic rats, respectively, indicating that AKBA stimulated glycolysis, glucose oxidation, and FA oxidation in the hearts of control and diabetic rats (Figure 3A–E). These effects of AKBA in diabetic LVs were prevented through pretreatment with CC.

### 3.3. AKBA Attenuated Weight Loss, Hyperglycemia, Hyperlipidemia, and Lipid Accumulation in the Heart and Liver of STZ Rats by Activating AMPK

There were no significant alterations (*p* > 0.5) in the fasting plasma, glucose, insulin, or serum levels of FFAs between the control and AKBA-treated rats (Table 1). However, serum, hepatic, and cardiac levels of CHOL and TGs, as well as serum levels of LDL-c were significantly reduced (*p* < 0.5) in AKBA-treated rats as compared to control group rats (Table 1). In contrast, the STZ-induced diabetic rats had a significantly higher body weight and showed a reduction in fasting insulin levels, with significantly higher levels of fasting glucose (*p* < 0.5) (Table 1). They also had significantly higher serum and hepatic levels of FFAs, TGs, and CHOL; serum levels of LDL-c; and cardiac levels of FFAs and TGs (*p* < 0.5) (Table 1). Body weights were significantly higher (*p* < 0.5) in STZ + AKBA-treated rats when compared to STZ-diabetic rats (Table 1). Furthermore, as compared to STZ-diabetic rats, STZ + AKBA-treated rats showed significantly fewer levels of fasting glucose, serum and hepatic FFAs, TGs, and CHOL; serum LDL-c; and cardiac TGs and CHOL (*p* < 0.5) (Table 1). However, no significant variations (*p* > 0.5) in the levels of any these markers were seen between the STZ-diabetic and STZ + AKBA + CC-treated rats, indicating that AKBA ameliorates hyperglycemia, hyperlipidemia, and hepatic/cardiac lipids by activating AMPK.

### 3.4. The Protective Effect of AKBA on Cardiac Structure and Function Required the Activation of AMPK

**Figure 4:**
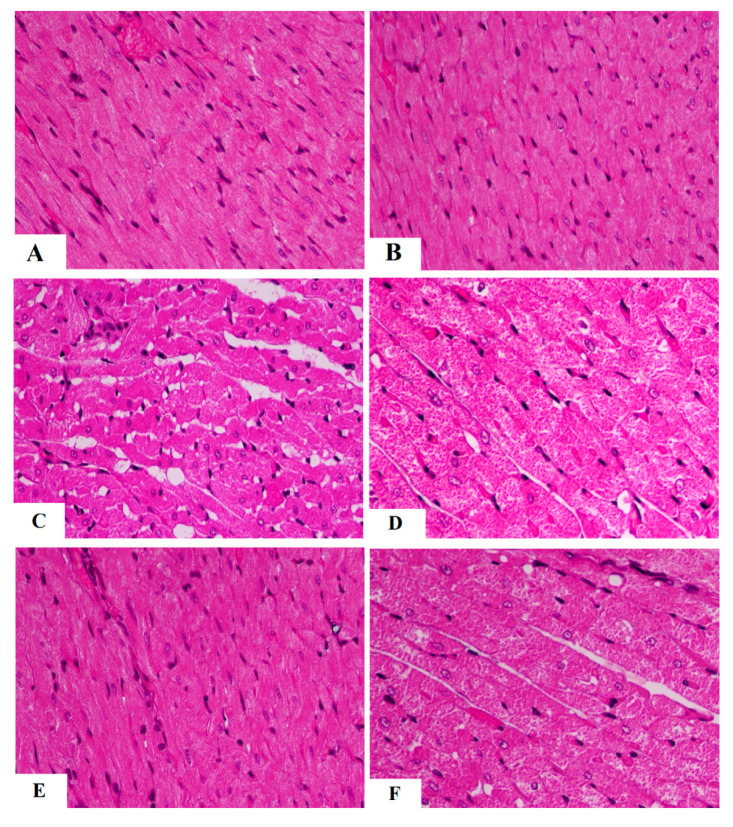
Morphological images of the left ventricles of all rat groups. (**A**,**B**) were taken from control and AKBA-treated rats and showed normal cardiomyocyte structure having normal striation (long arrow) and oval nuclei (short arrow). (**C**,**D**) were taken form STZ-diabetic LV and showed loss of muscle tissue (short arrow) with increased vacuolization (long arrow). Many nuclei were shrunk, abnormally round, and necrotic. (**E**) was taken from STZ + AKB-treated rats and showed almost normal morphology with normal cardiomyocytes and nuclei. (**F**) was taken from the LV of STZ + AKAB + CC-treated animals and showed similar loss of cardiomyocytes (arrow), increased vacuolization of the cardiomyocytes, and abnormally shrunk and round necrotic cells.

Control and AKBA-treated rats showed normal heart weights, and their LV showed intact striated branch muscle fibers with oval nuclei (Table 2 and Figure 4A,B). In addition, serum levels of troponin-I and CKMB and all measured cardiac hemodynamic parameters were not significantly different between these two control groups (*p* > 0.5) (Table 2). However, a significant increase in heart weight (*p* < 0.5) with obvious abnormal cardiac tissues and function was seen in STZ-treated rats as compared to the control group. In this regard, the myocardium of STZ-treated rats showed an obvious loss in the muscle fibers with increased vacuolization (Figure 4C). Moreover, most of the nuclei showed pyknosis, karyolysis, and karyorrhexis. In addition, STZ-diabetic rats had higher serum levels of troponin-I and CKMB, as well as LV levels of LVEDP, as compared to control rats (*p* < 0.5) (Table 2). Moreover, STZ-diabetic rats showed a significant reduction in LV levels of dp/dt_max_, dp/dt_min_, and LVSP, indicating impaired contractility and prolonged relaxation (*p* < 0.5) (Table 2). Heart weights, as well as serum levels of troponin-I and CKMB, were significantly reduced (*p* < 0.5) in STZ + AKBA-treated rats as compared to STZ-model rats, and their LV showed almost normal morphology (Table 2 and Figure 4D). They also showed significantly lower levels of LVEDP and higher values of dp/dt_max_, dp/dt_min_, and LVSP compared to the STZ-treated rats (*p* < 0.5) (Table 2). These effects afforded by AKBA were reversed after treatment with AMPK (Table 2 and Figure 4E,F). The heart morphology was similar, and heart weights and values of cardiac enzymes and hemodynamic parameters were not significantly different between the STZ-model rats and the STZ + AKBA + CC-treated rats. These data indicate that activation of AMPK is indispensable for the cardiac protection of AKBA.

### 3.5. AKBA Stimulated the Nrf2/Antioxidant Axis in the LV of the Control and STZ-Diabetic Rats in an AMPK-Dependent Manner

**Figure 5:**
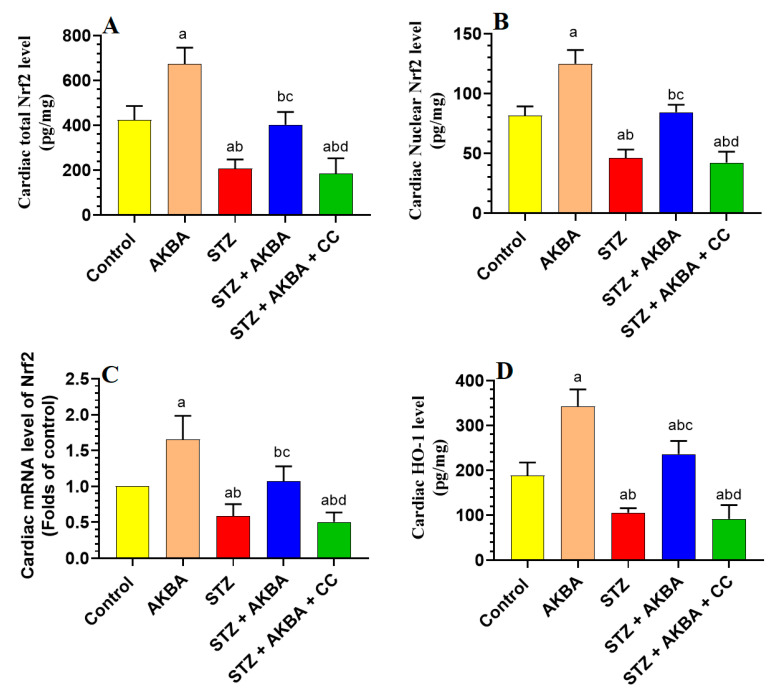
Total, nuclear, and mRNA levels of Nrf2 (**A**–**C**), and levels of heme oxygenase-1 (HO-1) (**D**) in the left ventricles (LVs) of all rat groups. Data were analyzed with one-way ANOVA followed by Tukey’s test. Values are presented as mean ± SD (n = 8/group). Data were considered significantly different at *p* < 0.5. a: significantly different as compared to control rats; b: significantly different as compared to AKBA-treated rats; c: significantly different as compared to STZ-diabetic rats; d: significantly different as compared to STZ + AKBA-treated rats. CC–compound C (selective AMPK inhibitor).

**Figure 6:**
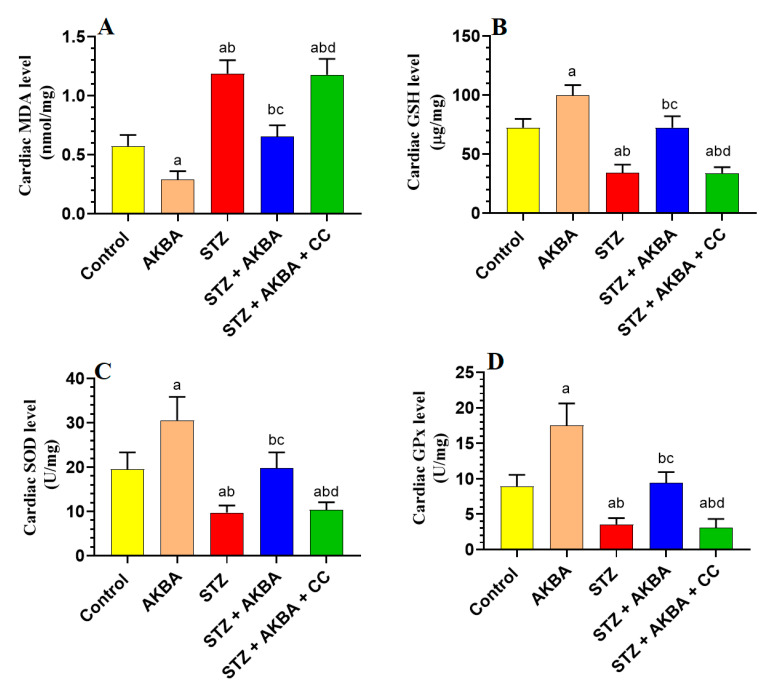
Total levels of malondialdehyde (MDA) (**A**), glutathione (GSH) (**B**), superoxide dismutase (SOD) (**C**), and glutathione peroxidase (GPx) (**D**) in the left ventricles (LVs) of all rat groups. Data were analyzed with one-way ANOVA followed by Tukey’s test. Values are presented as mean ± SD (n = 8/group). Data were considered significantly different at *p* < 0.5. a: significantly different as compared to control rats; b: significantly different as compared to AKBA-treated rats; c: significantly different as compared to STZ-diabetic rats; d: significantly different as compared to STZ + AKBA-treated rats. CC–compound C (a selective AMPK inhibitor).

mRNA, total, and nuclear levels of Nrf2, as well as levels of GSH, SOD, HO-1, and GPx, were significantly decreased, but MDA levels were significantly increased in the LVs of STZ-diabetic rats as compared to those of control rats (*p* < 0.5) (Figure 5A–D and Figure 6A–D). On the other hand, mRNA, total, and nuclear levels of Nrf2, as well as levels of GSH, SOD, HO-1, and GPx were significantly increased while MDA levels were significantly reduced in the LVs of both the AKBA- and STZ + AKBA-treated rats as compared to control and STZ-treated rats, respectively (*p* < 0.5) (Figure 5A–D and Figure 6A–D). However, a significant reduction in mRNA, total, and nuclear levels of Nrf2 and the levels of GSH, SOD, HO-1, and GPx with a significant increase in the levels of MDA was observed in the LVs of STZ + AKBA + CC-treated rats as compared to those of STZ + AKBA + CC-treated rats (*p* < 0.5) (Figure 5A–D and Figure 6A–D). These data indicate that AKBA stimulates the antioxidant capacity in the LVs of both the control and diabetic rats and depends on the activation of MAPK.

### 3.6. AKBA Suppressed NF-κB p65 and Cytokines Levels in the LVs of the Control and STZ-Diabetic Rats in an AMPK-Dependent Manner

**Figure 7:**
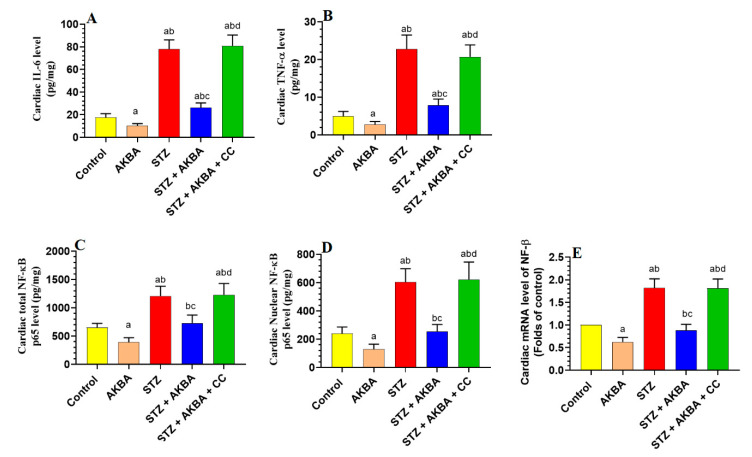
Total levels of interleukin-6 (IL-6) (**A**) and tumor necrosis factor-α (TNF-α) (**B**), as well as total, nuclear, and mRNA levels of NF-κB p65 (**C**–**E**) in the left ventricles (LVs) of all rat groups. Data were analyzed with one-way ANOVA followed by Tukey’s test. Values are presented as mean ± SD (n = 8/group). Data were considered significantly different at *p* < 0.5. a: significantly different as compared to control rats; b: significantly different as compared to AKBA-treated rats; c: significantly different as compared to STZ-diabetic rats; d: significantly different as compared to STZ + AKBA-treated rats. CC–compound C (a selective AMPK inhibitor).

LVs obtained from STZ-diabetic rats showed higher levels of TNF-α, IL-6, as well as mRNA, total, and nuclear levels of NF-κB p65, as compared to those of control rats (*p* < 0.5) (Figure 7A–E). The levels of TNF-α and IL-6, as well as mRNA, total, and nuclear levels of NF-κB p65, were significantly lower (*p* < 0.5) in the LVs of the control and STZ + AKBA-treated rats as compared to those of the control and STZ-treated rats (Figure 7A–E). These inhibitory effects of AKBA on the expression and nuclear translocation of NF-κB p65, as well as on the levels of TNF-α and IL-6 were diminished in the LVs of STZ + AKBA + CC-treated rats (Figure 7A–E).

### 3.7. AKBA Inhibited Intrinsic Cell Death in the LV of STZ-Diabetic Rats in an AMPK-Dependent Manner

**Figure 8:**
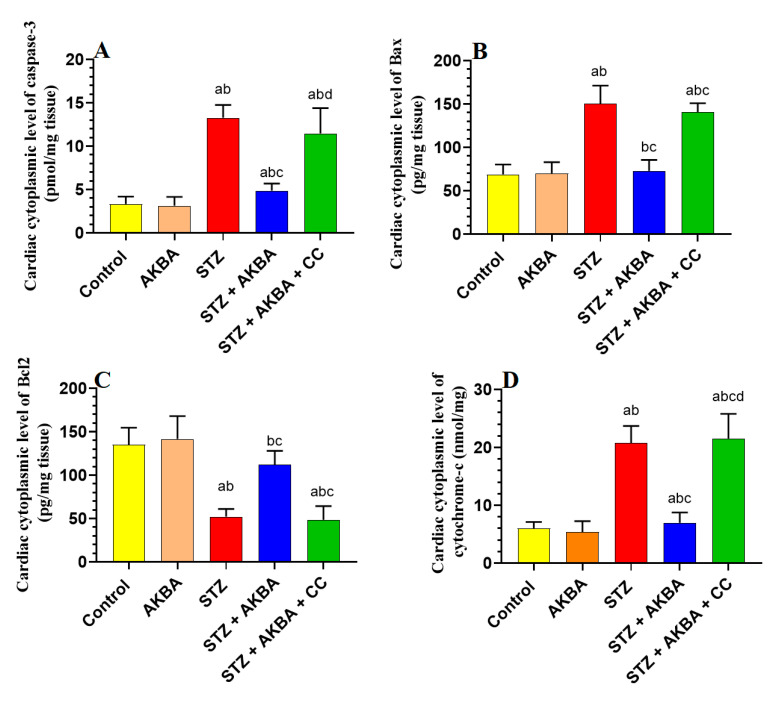
Cytoplasmic levels of caspase-3 (**A**), Bax (**B**), Bcl2 (**C**), and cytochrome-c (**D**) in the left ventricles (LVs) of all rat groups. Data were analyzed with one-way ANOVA followed by Tukey’s test. Values are presented as mean ± SD (n = 8/group). Data were considered significantly different at *p* < 0.5. a: significantly different as compared to control rats; b: significantly different as compared to AKBA-treated rats; c: significantly different as compared to STZ-diabetic rats; d: significantly different as compared to STZ + AKBA-treated rats. CC–compound C (a selective AMPK inhibitor).

No significant differences in the LV levels of Bax, caspase-3, cytochrome-c, or Bcl2 were seen between the control and AKBA-treated rats (*p* > 0.5) (Figure 8A–D). Levels of Bax, caspase-3, and cytochrome-c were significantly increased while levels of Bcl2 were significantly reduced in the LVs of STZ-diabetic rats as compared to those of control rats (*p* < 0.5). This pattern was significantly reversed (*p* < 0.5) in the LVs of STZ + AKBA-treated rats as compared to those of the STZ model rats (Figure 8A–D). The levels of all these markers were not significantly different between the STZ- and STZ + AKBA + CC-treated rats (*p* > 0.5) (Figure 8A–D). These results suggest that AKBA, by activating AMPK, inhibits the intrinsic pathway of apoptosis only in the LV of STZ-diabetic rats.

## 4. Discussion

The salient finding of this study revealed an interesting cardioprotective effect of AKBA against DC induced by STZ (T1DM) and illustrated some possible mechanisms of action. AKBA was able to preserve LV structure and systolic/diastolic functions of T1DM diabetic rats via acting centrally and peripherally and through interconnected mechanisms. These include (1) improving insulin levels, (2) attenuating hyperglycemia and hyperlipidemia, (3) restoring normal cardiac metabolism (i.e., stimulating glucose uptake and oxidation and FA oxidation), (4) activating the cardiac Nrf2/antioxidant axis and (5) inhibiting cardiac NF-κB p65/inflammatory cytokines axis. However, the most novel finding of this study is that activation of AMPK was evident as the major upstream mechanism by which AKBA acts to promote all the above-mentioned protective pathways. Indeed, suppressing AMPK with CC abolished the protective effect of AMPK on LV structures and function and prevented the hypoglycemic and hypolipidemic, as well as the cardiac metabolic, antioxidant, and anti-inflammatory protective effect of AKBA in these STZ-diabetic rats.

During the early stages of DCM, metabolic disturbance of glucose and FA metabolism due to hyperglycemia, insulin deficiency/resistance, hyperlipidemia, and increased uptake of nonesterified FA promote cardiac dysfunction through inducing oxidative stress, maladaptive immune modulation, and inflammation [53,54]. These events are associated with fibrotic diastolic dysfunction and reduced ejection fraction [53]. During the later stages of DCM, the alterations in cardiac structure and function are more pronounced due to the increase in cardio myocyte apoptosis, microcirculatory dysfunction, intestinal fibrosis, cardiac hypertrophy, and capillary microaneurysms [53,55,56]. At this stage, there is an impairment in both diastolic and systolic dysfunction [53]. In this regard, with the absence of insulin signaling and subsequent lack of glucose uptake, there is a reduction in the activity of Ca2+ ATPase, which subsequently reduces cytoplasmic levels of Ca2+ by increasing its uptake by the sarcoplasmic reticulum [53]. In addition, the impairment of insulin signaling impairs coronary NO-dependent vasodilation, which can also suppress cardiac contractility and promote cardiac apoptosis/necrosis [53]. On the other hand, hyperglycemia by itself can induce glucotoxicity and stimulate the protein glycation and the production of advanced glycation end products (AGEs), which in turn contribute to cardiac stiffness and impairment of diastolic relaxation by increasing connective tissue crosslinking and promoting ROS production, oxidative stress, and inflammation [53,57]. Nonetheless, the higher uptake of FFA uptake reduces glucose (expression of GLUT4), impairs insulin signaling and uptake, and promotes cardiac lipotoxicity, oxidative stress, and inflammation by increasing levels of TGs, CHOL, and other lipid metabolites such as diacylglycerol (DAG) and ceramides [58]. However, insulin injections, as well as stimulating insulin release or reducing circulatory glucose and lipid levels with hypoglycemic and hypolipidemic agents, have been shown to protect against DCM in diabetic subjects and animal models with T1DM and T2DM [3,10,56,59].

In this study, our data confirmed the cardiac protective effect of AKBA in STZ-diabetic and insulin-deficient rats. Within this view, AKBA was not only able to restore normal LV histological features but was also able to improve the LV systolic and diastolic functions. This protection was also associated with potent hypolipidemic and antihyperglycemic effects, which in turn, reduced hepatic, cardiac, and serum levels of TGs, CHOL, and FFAs. These data support the previous observations by others who have also demonstrated the hypoglycemic and hypolipidemic effects of AKBA and other extracts of *B. serrata* in animal models of T1DM and T2DM [34,35]. In addition, AKBA also reduced cardiac and hepatic levels of FFAs, CHOL, and TGs, and reduced serum levels of CHOL, TGs, and LDL-c while increasing serum levels of HDL-c in the control rats. Therefore, it is reasonable to assume that AKBA exerts a potent hypolipidemic effect by suppressing hepatic de novo lipogenesis and reducing the cardiac and hepatic uptake of FFAs. In addition, treatment with AKBA also reduced fasting hyperglycemia and partially increased circulatory insulin levels in these diabetic rats. Hence, it can be concluded that the cardiac protective effect of AKBA observed in this study is partially due to the hypoglycemic, insulin-releasing, and hypolipidemic effects of AKBA, which together have a beneficial impact on the heart as discussed above. However, since AKBA was unable to modulate circulatory FFAs, glucose, and insulin levels in control-treated rats, these data indicate that AKBA has an antihyperglycemic effect that is not related to modulating hepatic gluconeogenesis or stimulating insulin release but rather due to improving peripheral glucose uptake and enhancing peripheral insulin sensitivity. However, this could be due to the ability of AKBA to partially recover the function of pancreatic β-cells, possibly by suppressing STZ-induced oxidative stress, inflammation, and apoptosis. Indeed, it has been previously shown that treatment with IKBA and extracts from *B. serrata* stimulates insulin release from the survival of pancreatic β-cells through stimulating antioxidants (e.g., SOD, CAT, and GSH) and suppresses macrophage infiltration and NF-κB-mediated inflammation [36,37,55]. In addition, such an increase in insulin levels could also be indirectly responsible for the hypoglycemic effect of AKBA via the suppression of hepatic gluconeogenesis [60].

An interesting observation in this study was that treatment with CC completely prevented the hypoglycemic, hypolipidemic, and insulin-releasing effects of AKBA in the STZ-diabetic rats. Therefore, these metabolic effects of AKBA can be assumed to be AMPK-dependent. Indeed, hyperglycemia has been consistently associated with reduced levels of cardiac, hepatic, and adipose tissue activities of AMPK [17,18,19,61,62]. On the other hand, the activation of AMPK by direct and indirect activators, as well as plant-derived activators, could alleviate obesity, hyperglycemia, IR, hepatic lipotoxicity, and hyperlipidemia in animal models of obesity and metabolic syndrome [61,62,63]. It has also been shown to reduce plasma glucose levels, attenuate hyperlipidemia, and protect against renal damage in STZ-treated rats [64,65]. Within this view, the hypolipidemic effect of AMPK can be attributed to its phosphorylation capabilities which result in the inhibition of lipogenesis and the stimulation of the oxidation of FAs by suppressing SREBP1, FA-synthesis genes, and ACC [66]. On the other hand, AMPK can reduce fasting glucose levels and enhance IR by stimulating peripheral glucose clearance and increasing the membranous translocation of GLUT2/4, and lowering membrane cholesterol [13,67,68]. Furthermore, the activation of AMPK by metformin, AICAR, and a-769662 has been shown to suppress hepatic gluconeogenesis and glucose production by reducing the expression and activation of key glucose-synthesis enzymes such as PEPCK and G6Pase activity in STZ-diabetic mice [66,69]. These drugs, and in particular metformin, also inhibited gluconeogenesis by antagonizing glucagon signaling and suppressing lipid synthesis [70].

Metformin can also reduce fasting hyperglycemia by decreasing bile acid absorption and increasing glucagon-like protein-1 (GLP-1) [71]. Interestingly, other studies have also shown that the activation of AMPK can improve β-cell function, mitigate the apoptotic pathways, and restore β-cell oxidative metabolism to stimulate insulin secretion. Indeed, the activation of AMPK through metformin was reported to reduce fasting glucose levels and prevent diabetic nephropathy in STZ-diabetic rats [72]. In addition, treatment with AICAR can prevent apoptosis and improve the insulin-releasing function of pancreatic β-cells without changing lipid levels [73,74]. Similarly, activation of AMPK with berberine was shown to stimulate the release of insulin from pancreatic rats’ islets by decreasing ATP production and O2 consumption [75]. Moreover, AMPK is a potent antioxidant and anti-inflammatory molecule that can stimulate pancreatic cell survival by inducing autophagy, promoting mitochondria biogenesis, stimulating antioxidant genes, and suppressing inflammation [17,76].

In addition, cardiac substrate metabolism is a determinant factor for cardiomyocyte health. Under normal conditions, insulin stimulates glucose uptake, glycolysis, and glucose oxidation while increasing FA uptake and inhibiting FA oxidation [3]. In this regard, insulin stimulates glucose uptake by increasing the expression of GLUT4 and enhances glucose oxidation by stimulating the mitochondria PDH complex [3,10]. Malonyl CoA, produced by the ACC enzyme, is a natural inhibitor that suppresses FA mitochondria transport and oxidation by reducing the expression and activities of the expression of CPT1 [77]. Insulin also stimulates glycolysis, thus increasing the levels of citrate, which can further inhibit FA oxidation by increasing the levels of malonyl CoA levels [77]. In the absence of insulin, FFAs are the preferred heart fuel, which can lead to reduced cardiac contractility and cardiac injury by suppressing glucose usage and stimulating ROS and inflammatory cytokine production [3]. Indeed, the higher uptake and oxidation of FFAs suppress glycolysis by activating pyruvate dehydrogenase kinase (PDK), which in turn suppresses glycolysis and glucose oxidation by inhibiting PFK-1 PDH, respectively [10]. In this investigation, our data also indicated that FFAs levels and oxidation were significantly increased in the LVs of STZ-treated hearts and were associated with the reduced utilization and oxidation of glucose as shown by the reduced activities of ACC1, PFK1, and PDH, as well as in the levels of malonyl CoA. On the other hand, AKBA was able not only to restore normal cardiomyocyte glucose utilization and oxidation but also enhance fatty acid oxidation in the hearts of both the control and STZ-treated rats. Indeed, treatment with AKBA not only reversed the reduction in the activities of PDH and PFK-1, but also inhibited the activities of ACC, reduced levels of malonyl CoA, and further stimulated levels of CPT1. Even this could be explained by the higher insulin levels in the STZ-diabetic rats post-AKBA. Similar effects on all markers were also seen in the LVs of control rats, also suggesting an insulin-independent mechanism. Based on this, we have concluded that AKBA has stimulatory effects on glycolysis, glucose oxidation, and FA uptake and oxidation under basal and diabetic conditions. Indeed, we have found that the ability of AKBA to stimulate cardiac glucose metabolism is AMPK-dependent, as treatment with CC abolished all the stimulatory effects of AKBA on these glucose-metabolism-related markers. This is the first evidence in the literature concerning the novel role of ALBA I regulation of cardiac metabolism through the activation AMP, which could lead to the development of new therapies. As evidence of this, AMPK was reported to stimulate the cardiac uptake of glucose through the translocation of GLUT-4 to the sarcolemma via acting through several pathways [68,78]. In addition, AMPK can stimulate glycolysis by suppressing mTOR signaling and through the upregulation and phosphorylation-induced activation of PFK-1 [79,80,81,82]. It can also stimulate the cardiac uptake of FFAs by increasing the sarcolemma expression of CD36 [80,82]. Furthermore, AMPK stimulates CPT1 levels and FA oxidation via the phosphorylation inhibition of ACC [80,82]. Together, these data are very similar to our findings and further confirm our hypothesis.

Oxidative stress and inflammation are the major mechanisms underlying DCM [83,84]. In the majority of cells, including the cardiomyocytes, Nrf2 is the major antioxidant transcription factor that enhances cell survival and inhibits oxidative stress and inflammation by stimulating the synthesis of GSH and increasing the expression of antioxidant genes such as SOD, CAT, glutathione peroxidase (GPx), and heme-oxygenase-1 [85]. On the other hand, NF-κB is the major inflammatory transcription factor that initiates cell inflammation by increasing the transcription and the release of inflammatory cytokines [86]. In diabetic hearts, there is a sustained increase in the production of ROS and inflammatory cytokines and adhesive molecules. This could be due to various mechanisms related to abnormal glucose and FFA metabolism and has been explained in other excellent studies and reviews [83,84]. In addition, the sustained activation of NF-κB is associated with a reduction in the expression and activities of Nrf2, both of which further stimulate cardiac damage. On the other hand, both cytokines and ROS trigger cardiomyocyte mitochondria-mediated (intrinsic) apoptosis and fibrosis by increasing the expression of apoptotic markers (p53, Bax, caspases 3/9) and collagen synthesis from fibroblasts [87,88]. However, alleviating oxidative stress, inflammation, activation of Nrf2, or suppression of NF-κB has been found to protect against DM-induced cardiac damage, fibrosis, and apoptosis [84,85,89,90]

We further discovered that AKBA could also act by a third insulin-independent but AMPK-dependent antioxidant and anti-inflammatory mechanism that involves the upregulation of the Nrf2/antioxidant axis and suppression of the NF-κB/inflammatory signaling pathway. In accordance with this, we found that AKBA was able to stimulate the expression and activities of Nrf2 and to increase the levels of SOD, GSH, CAT, and HO-1 in the hearts of both the control and diabetic rats, suggesting a stimulatory effect on the Nrf2/antioxidant axis. In the same manner and in both treated groups, AKBA was able to alleviate the elevated expression and activities of NF-κB, which indicates the presence of direct inhibitory effects. However, as AKBA was only able to attenuate the increase in the levels of p53, caspase-3, and cytochrome-c in the LVs of diabetic rats but not in those of hearts of control rats, we become confident that the antiapoptotic effect of AKBA is secondary to its antioxidant and anti-inflammatory effects. In addition, as treatment with CC abolished the effects of AKBA on the regulation of Nrf2 and NF-κB, these data suggest that these effects are AMPK-dependent. The regulation of AKBA on Nrf2 and NF-κB also supports many other previous studies in other animal models. In this regard, boswellic acid was reported to prevent doxorubicin-induced hepatotoxicity and liver damage in rats by upregulating and stimulating the Nrf2/HO-1 axis [91]. In addition, dietary supplementation with *B. serrata* gum powder was shown to prevent liver and pancreatic damage in STZ-treated rats by suppressing lipid peroxidation and stimulating GSH, SOD, and CAT [36]. In addition, it has been shown to prevent UV-mediated skin damage, ethidium bromide-induced multiple sclerosis, hydrogen peroxide-induced retinal damage, and amyloid-β-induced cognitive impairment and brain damage by upregulating/activating the Nrf2/HO2/antioxidant axis [92,93,94]. Moreover, in vitro, experimental, and clinical studies have confirmed the ability of IKAB and other extracts of *B. serrata* to ameliorate psoriasis, atherosclerosis, asthma, colitis, rheumatoid arthritis, and osteoclastogenesis by suppressing NF-κB and inflammatory cytokine production [95,96,97,98], a phenomenon reviewed by Ammon [34]. In addition to this, accumulating data have shown that AMPK attenuates oxidative stress and inflammation in several disorders by activating the Nrf2/antioxidant axis and suppressing the NF-κB/inflammatory signaling pathway [99,100]. The precise molecular mechanisms by which AMPK regulates Nrf2 and NF-κB have been established. Within this view, previous studies have documented the ability of AMPK to stimulate Nrf2 signaling at the mRNA level [101]. In addition, AMPK was found to suppress the nuclear export of Nrf2 by activating the Akt-induced suppression of the GS3K/Fyn signaling pathway [24]. Furthermore, AMPK can directly stimulate the nuclear translocation of Nrf2 by direct phosphorylation at site Ser558 [102]. However, AMPK has no phosphorylation site on NF-κB. Instead, AMPK can inhibit the transcription, phosphorylation, and nuclear translocation of NF-κB by acting on several downstream regulators such as SIRT1, Nrf2, and Forkhead box O (FoxO) factors [103].

### Conclusions, Study Limitations, and Future Studies

Through this study, we report the first evidence of AKBA as a novel molecule that can alleviate DCM in STZ-diabetic rats. In addition, we demonstrated that this protection is mediated by AMPK-mediated antihyperglycemic, hypolipidemic, antioxidant, and anti-inflammatory effects, as well as the regulation of the cardiac glucose/lipid metabolism. Despite these data, this study still has some limitations. Most importantly, these data remain observational. Therefore, further studies using AMPK-deficient cells or animals could provide further insights to confirm these findings. In addition, the regulation of AMPK is a very complicated process, which includes several upstream targets including microRNA, kinases, sirtuin-1 (SIRT1), and others. Therefore, completing further studies to determine the major upstream regulator induced by AKBA is a priority. In addition, more studies targeting the effect of AKBA on other animal models, such as T2DM, as well as the effect of AKBA on adipose tissue and muscle transcriptional factors regulating glucose and lipid metabolisms, could provide a piece of further evidence concerning the mechanism of action of this molecule.

## Figures and Tables

**Table 1:** Levels of biochemical markers in the serum and plasm of rats of all groups.

		Control	ABKA	STZ	STZ + ABKA	STZ + ABKA + CC
	Final body weight	411.5 ± 36.7	421.5 ± 31.2	301 ± 25.7 ^ab^	398 ± 31.3 ^abc^	318 ± 29.2 ^abd^
Plasma	Glucose (mg/dl)	106.3 ± 9.3	102.1 ± 8.2	341 ± 33.2 ^ab^	143 ± 12.5 ^abc^	328 ± 27.8 ^abd^
	Insulin (ng/mL)	4.7 ± 0.7	4.4 ± 0.5	2.1 ± 0.3 ^ab^	3.25 ± 0.4 ^abc^	2.18 ± 0.3 ^abd^
Serum	TGs (mg/dl)	66.4 ± 4.3	52.5 ± 45 ^a^	137 ± 11.2 ^ab^	79.4 ± 6.8 ^abc^	129 ± 11.4 ^abd^
	CHOL (mg/dl)	78.4 ± 7.9	59.7 ± 6.6 ^a^	168 ± 15.6 ^ab^	86.8 ± 6.1 ^abc^	183 ± 4.5 ^abd^
	LDL-c (mg/dl)	44.1 ± 5.8	31.2 ± 3.7 ^a^	97.6 ± 8.4 ^ab^	51.8 ± 5.9 ^abc^	104 ± 9.3 ^abd^
	FFAs (μmol/mg)	398 ± 43.2	376.1± 29.8	893.2 ± 77.3 ^ab^	484.2 ± 36.8 ^abc^	991 ± 86.5 ^abcd^
Liver	TGs (mg/g)	5.8 ± 0.7	4.1 ± 0.5 ^a^	11.3 ± 1.6 ^ab^	7.1 ± 0.9 ^abc^	12.4 ± 2.7 ^abd^
	CHOL (mg/dl)	4.9 ± 0.39	3.1 ± 0.4 ^a^	8.8 ± 0.6 ^ab^	5.1 ± 0.4 ^abc^	9.3 ± 0.9 ^abd^
	FFAs (μmol/mg)	97.5 ± 8.4	76.2 ± 6.4 ^a^	388.1 ± 29.6 ^ab^	129 ± 11.3 ^abc^	369.2 ± 26.8 ^abd^
Heart	TGs (μg/g)	50.4 ± 5.1	36.7 ± 3.5 ^a^	111.4 ± 9.7 ^ab^	61.3 ± 5.7 ^abc^	119.4 ± 10.1 ^abd^
	FFAs (nmol/mg)	123.2 ± 11.3	88.2 ± 7.3 ^a^	365.3 ± 22.5 ^ab^	162.2 ± 13.8 ^abc^	389.9 ± 33.5 ^abd^

Values are presented as mean ± SD (n = 8/group). Data were considered significantly different at *p* < 0.5. a: significantly different as compared to control rats; b: significantly different as compared to AKBA-treated rats; c: significantly different as compared to STZ-diabetic rats; d: significantly different as compared to STZ + AKBA-treated rats. CC–compound C (a selective AMPK inhibitor).

**Table 2:** Levels of cardiac enzymes and markers of left ventricular (LV) function in the rats of all groups.

		Control	ABKA	STZ	STZ + ABKA	STZ + ABKA + CC
	Heart weight (g)	1.2 ± 0.2	1.15 ± 0.18	1.46 ± 0.31 ^ab^	1.14 ± 0.27 ^abc^	1.52 ± 0.5 ^abd^
Serum	Troponin-I (pg/mL)	76.3 ± 6.5	71.7 ± 7.5	583 ± 42.5 ^ab^	112.5 ± 11.9 ^abc^	612 ± 58.3 ^abd^
	CK-MB (pg/mL)	212.1 ± 18.7	209.5 ± 20.4	633.4 ± 54.9 ^ab^	298.6 ± 25.4 ^abc^	618 ± 52.2 ^abd^
LV function	dp/dt_max_ (mmHg)	5994 ± 439	5778 ± 593	2934 ± 209 ^ab^	4938 ± 398 ^abc^	2609 ± 211 ^abd^
	dp/dt_min_ (mmHg)	4983 ± 375	4819 ± 478	2022 ± 199 ^ab^	3772 ± 289 ^abc^	2231 ± 245 ^abd^
	LVSP (mmHg)	122.4 ± 11.3	125.1± 12.7	75.3 ± 6.9 ^ab^	108.5 ± 9.9 ^abc^	69.6 ± 5.8 ^abd^
	LVEDP (mmHg)	2.9 ± 0.3	3.1 ± 0.3	11.5 ± 1.6 ^ab^	3.7 ± 0.4 ^abc^	13.1 ± 2.7 ^abd^

Values are presented as mean ± SD (n = 8/group). Data were considered significantly different at *p* < 0.5. a: significantly different as compared to control rats; b: significantly different as compared to AKBA-treated rats; c: significantly different as compared to STZ-diabetic rats; d: significantly different as compared to STZ + AKBA-treated rats. CC–compound C (a selective AMPK inhibitor).

## Data Availability

The datasets used and analyzed in the current study are available from the corresponding author upon reasonable request.
